# Genome-wide characterization of extrachromosomal circular DNA in gastric cancer and its potential role in carcinogenesis and cancer progression

**DOI:** 10.1007/s00018-023-04838-0

**Published:** 2023-06-27

**Authors:** Xianming Jiang, Xiaoguang Pan, Wenchao Li, Peng Han, Jiaying Yu, Jing Li, Haoran Zhang, Wei Lv, Ying Zhang, Yulong He, Xi Xiang

**Affiliations:** 1 Scientific Research Center, The Seventh Affiliated Hospital, Sun Yat-Sen University, Shenzhen, 518107 Guangdong China; 2 Department of Biology, University of Copenhagen, 2200 Copenhagen, Denmark; 3 Qingdao-Europe Advanced Institute for Life Sciences, BGI-Qingdao, Qingdao, 266555 Shandong China; 4 College of Medicine and Forensics, Xi’an Jiaotong University Health Science Center, Xi’an, 710061 Shanxi China; 5 College of Life Sciences, University of Chinese Academy of Science, Beijing, 100049 China; 6 Guangdong Provincial Key Laboratory of Digestive Cancer Research, The Seventh Affiliated Hospital, Sun Yat-Sen University, Shenzhen, 518107 Guangdong China; 7 Tomas Lindahl Nobel Laureate Laboratory, The Seventh Affiliated Hospital, Sun Yat-sen University, Shenzhen, 518107 Guangdong China

**Keywords:** EcDNA, Double minutes, DM, MicroRNA, Genome remodeling, Rolling circle amplification

## Abstract

Extrachromosomal circular DNAs (eccDNAs) carrying random genomic segments are broadly found across different cancer types, but their molecular functions and impact in gastric cancer (GC) are rarely known. In this study, we aimed to investigate the potential role of eccDNA in GC. Using the Circle-seq strategy, we observed the eccDNA abundance in gastric cancer tissues (GCT) was aberrantly higher than that of normal adjacent tissues (NAT). The high abundance of eccDNAs carrying oncogene-segments in GCT may represent the DNA damage products of amplified oncogenes. Analysis of GCT over-represented eccDNA carrying enhancer (eccEnhancer) based on data from FANTOM5 project combined with TCGA database suggested the GC over-represented eccEnhancers may contribute to development of GC. GC over-represented eccDNAs carrying pre-miRNA (eccMIR) were enriched to multiple cancer-relevant signal pathways by KEGG analysis. We then synthesized the top six GC over-represented eccMIRs and found four of them enabled high expression of miRNAs and down-regulation of miRNA-target genes in MGC803 cells. Furthermore, we observed the inheritance of GC over-represented eccMIRs benefited host cell proliferation and promoted the aggressive features of host cells. Altogether, this study revealed the GC over-represented eccDNAs carrying functional genomic segments were related to the carcinogenesis of GC and presented the capability to facilitate cancer progression, suggesting the cancerous eccDNAs may serve as a dynamic reservoir for genome plasticity and rapid adaptive evolution of cancer. Therefore, blocking the pathways for eccDNAs generation may provide a novel therapeutic strategy for the treatment of gastric cancer.

**Supplementary Information:**

The online version contains supplementary material available at 10.1007/s00018-023-04838-0.

## Introduction

The functional and numerical diversity of eccDNA confers its powerful roles in the rapid remodeling of cancer genome and driving intratumoral heterogeneity [1]. Most recent studies focus on the large size eccDNA carrying intact oncogene (known as “ecDNA”), as the focal amplification of oncogene on ecDNA is proved to be a driver for tumorigenesis and cancer evolution [2–5]. In addition, ecDNA carrying regulatory elements, such as an enhancer, can shape the oncogene amplification via intramolecular interactions [6, 7]. Moreover, clusters of ecDNAs can form ecDNA hubs and mediate dense intermolecular contacts between ecDNA and chromosomal DNA, thereby driving genome-wide transcriptional amplification [4, 8].

The story of eccDNAs in cells is that most of them will be lost during cell mitosis, due to they lack a centromere [9]. Only those that provide host cells with adaptive advantages can survive [10]. Therefore, the inheritance pattern of ecDNA in tumor cells is dynamically random under neutral selection, while environmental or treatment challenges can cause certain ecDNA to increase rapidly in copy number [11]. In some cases, “lucky” eccDNAs can integrate into the genomic DNA [12], providing stable survival advantages to hosts and the daughter cells. These characteristics of eccDNA make it capable of remolding genomes and promoting rapid adaptive evolution in cancer cells [1].

Applications of high-throughput sequencing technologies and advanced bioinformatic algorithms allow the discovery of tens of thousands of eccDNAs [2, 13–16]. Beyond the mega-base-pairs ecDNA, it is becoming increasingly difficult to ignore the huge number of relatively small-size eccDNAs. The majority of eccDNA in cells or tissues detected by next-generation or long-read Nanopore sequencing is less than 10 kilo-base-pairs (kb) [16–19]. These eccDNAs carrying random and short genomic segments with enigmatic functions are recognized as products of DNA damage [17] or genome rearrangement [20]. A limited study has shed light on the regulatory functions of small eccDNA harboring miRNA gene or exonic sequence [21], but its association with GC and impact on cancer is unknown.

Herein, we performed a comprehensive analysis of the genome-wide eccDNA profiles of GCT and NAT derived from GC patients, including the general eccDNA features and the relevance of GCT over-represented eccDNAs, which carry different functional genomic segments, to GC. In addition, we demonstrated the molecular functions of GCT over-represented eccMIRs as miRNA producers in MGC803 cells and found they promoted the proliferation and aggressive features of host cells in a transient manner. Based on the findings of our work, we proposed the theoretical hypothesis of how the eccDNAs evolve cooperatively with cancer cells and therefore, blocking the generation of eccDNAs may provide a potential therapeutic strategy for the treatment of GC promoted by abnormal production of eccDNAs.

## Materials and methods

### GC patients recruitment and samples collection

Thirteen patients with gastric cancer (median age 63 years [range from 37 to 79 years], 11 males/2 females) were recruited from the Seventh Affiliated Hospital of Sun Yat-sen University. All the patients in this study were not treated with chemotherapy or radiotherapy before surgery. They have signed an informed consent form proved by the Institutional Review Board (IRB) of Seventh Affiliated Hospital of Sun Yat-sen University before the sampling. The cancer tissues and matched normal adjacent tissues were preserved with snap freezing in liquid nitrogen and then transferred to a − 80 °C refrigerator for long-term storage.

### eccDNA purification and RCA amplification

The eccDNA of the tissue sample was purified and amplified as previously described with some modifications [22]. Briefly, the tissue samples (~ 10 mg) were chopped into small pieces in a cell culture plate and the genomic DNA was purified by the MagAttract HMW DNA Kit (Qiagen). 900 ng genomic DNA of each sample was input for linear-DNA removal by the plasmid-safe ATP-dependent DNase (PSD) (Epicenter) in a 50 µL reaction system. Two spike-in plasmids and a 7 kb linear-DNA fragment amplified from a cas9 plasmid were added into the reaction mixture and acted as the external circular- and linear-DNA references, respectively. The reactions were carried out at 37 °C continuously for 7 ~ 12 days and added additional ATP and PSD every 24 h according to the manufacturer’s protocol. Standard PCR on the internal (Cox5b gene) and external references (spike-in plasmids and 7 kb linear fragment) was used to evaluate the removal of linear DNA and retention of circular DNA in each sample (Fig. S1A and B). Samples with no Cox5b and 7 kb linear PCR bands were subjected to the subsequent Rolling Circle Amplification (RCA). The purified eccDNA was amplified using the Phi29 polymerase (Thermo Scientific) at 30 °C continuously for 48 h according to the manufacturer’s protocol. All the DNA purification was performed by the DNA clean beads (Vazyme).

### library preparation and sequencing

About one ug of Phi-29 RCA amplified DNA was sheared by sonication (Covaris LE220) to generate a median size of 400 bp. Then, 500 ng of fragmented DNA was input for library construction by MGIEasy Library Preparation Kit (MGI-BGI, China). Each library’s length distribution and quality were examined by the Bioanalyzer 2100 (Agilent). A pool of two types of tissue samples (GCT and NAT) was sequenced as 2 × 150-nucleotide paired-end reads on one lane (BGI DNBseq T5).

### eccDNA calling by circle-map

To detect eccDNA from CircleSeq data, we use the same method described in [23]. In detail, reads were cleaned for both quality and potential adapter sequences. Burrows-Wheeler Aligner MEM v0.7.15 with default parameters was used to align the paired reads to the human reference genome GRCh38 (UCSC). The aligned BAM files were then sorted both by sequence name and genomic coordinate. The above files were used as input files for Circle-Map +  + v1.0.0 to detect the exact eccDNA regions.

To determine the true positive of real circles versus background noise, some filtering parameters were used as follows: (1) The split reads ≥ 2; (2) circle score ≥ 200; (3) coverage increase in the start coordinate ≥ 0.33; (4) coverage increase in the end coordinate ≥ 0.33; (5) coverage continuity = 0.2; (6) The SD of coverage is smaller than the mean coverage over the whole eccDNA region, and (7) If the length of eccDNA is larger than 2 K, the eccDNA should have more than one discordant reads.

### eccDNA annotation and analysis

We used the protein-coding annotation of the GRCh38 downloaded from Ensembl BioMart. Overlaps between detected eccDNA coordinates and annotated genes were found using bedtools (iintersect-wo). Custom R scripts were used to filter the annotated eccDNA genes by keeping the overlapped base pair numbers greater than 60. We also calculated the junction count for each gene, which we called eccDNA abundance. To make every sample comparable, all the samples were normalized by the length of the gene and the total library size. First, we calculated the eccGene counts of a certain gene by “eccGene count/gene length”. Second, to eliminate the bias introduced by NGS depth, the value of “eccGene counts/gene length” was divided by the total reads number obtained in the sample, which we termed as “eccGene counts per million reads mapped” (CPM). Third, the CPM of certain eccGene was normalized again by “certain eccGene CPM/total of CPM value” to obtain the normalized eccGene count ratio.

### PCR validation of candidate eccDNA

Validation of the eccDNA of interest was performed by outward-, inward-PCR and Sanger sequencing of the junction site and internal DNA region. Each 20 µL PCR reaction consisted of 0.2 µL RCA product, 0.6 µL forward primer (10 µM), 0.6 µL reverse primer (10 µM), 12.5 µL 2 × Rapid Taq Master Mix (Vazyme) and supplemented with ddH_2_O. The thermocycling PCR program was 95 °C for 2 min, (95 °C for 15 s, 55 °C for 15 s, 72 for 5 s) with 35 cycles, 72 °C for 5 min and 4 °C hold. PCR primers for eccEnhancers and eccMIRs validation are listed in Supplemental Table S4 and Table S6, respectively.

### eccDNA synthesis by LAMA

The synthetic eccDNAs were prepared using the LAMA strategy as previously described [21]. The 1 kb Random DNA sequence in the eccRandom with 50% guanine–cytosine content was generated by the webtool of “Random DNA sequence generator” (http://www.faculty.ucr.edu/~mmaduro/random.htm). Then the half-complemental linear-DNA fragments of each eccDNAs were synthesized by GenScript and their PCR amplification primer sets were synthesized in BGI. All the synthetic linear-DNA fragments (Linear A and B for one eccDNA) and the matched primers are listed in Supplemental Table S7. For LAMA reaction, equal amounts of the linear A and B amplification products were mixed with the Taq DNA ligase (NEB) and buffer. The reaction was performed in the thermocyclers with 95 °C for 5 min, (95 °C for 20 s, 4 °C for 1 min and 65 °C for 20 min) with 10 cycles. The LAMA reaction product was then purified with DNA clean beads (Vazyme) and digested with Exonuclease V (NEB) to remove residual linear DNAs. The final purified LAMA DNA circles were digested by appropriate restriction enzymes (RE) for circular-structure verification. Only those that had one linear band after single RE digestion were used for subsequent experiments.

### MGC803 cell culture and transfection with synthetic eccDNA

The human gastric cancer cells MGC803 (ATCC) were cultured in RPMI 1640 culture medium supplemented with 10% fetal bovine serum (FBS) (Gibco) and 1% penicillin/streptomycin (Invitrogen) at 37 °C with 5% CO_2_ and maximum humidity. The transfection was conducted using the lipofectamine2000 (Invitrogen) in 24-well plate following the manufacturer’s instruction. Briefly, 60,000 cells were seeded in a well of the 24-well plate one day before transfection. For each transfection, the 50 µL mixture consisted of 500 ng synthetic eccDNA, 0.75 µL lipofectamine2000 and supplemented with opti-M (Invitrogen). Each transfection in one group was performed in at least triplicate. The cells were harvested for subsequent analysis 48 h after transfection. In this study, transfection of eccRandom was used as the control group for all experiments.

### qPCR assay of miRNA and target mRNA

We performed qPCR assay for detection of the miRNA and target mRNA expression level. The synthetic eccDNA transfected cells were harvested 2 days after transfection and the total RNAs were purified using RNA extraction reagent (SeviceBio) according to the manufacturer’s instruction. 1 µg RNA was input for reverse-transcription (RT) and miRNA cDNA was synthesized using miRNA 1st Strand cDNA synthesis kit (by stem-loop) (Vazyme). We used U6 as the internal reference gene and both the target miRNA and U6 were created in the same RT reaction. The qPCR of miRNA was conducted in replicate using the miRNA Universal SYBR qPCR Master Mix (Vazyme). Primers for miRNA and U6 cDNA RT and qPCR are listed in supplemental Table S8. The cDNA of total RNA was created using HiScript III 1st strand cDNA synthesis kit (+ gDNA wiper) (Vazyme) and qPCR was performed in replicate using the ChamQ Universal SYBR qPCR Master Mix (Vazyme). qPCR primers for target mRNAs were listed in Supplemental Table S9. The relative expression level of miRNA and mRNA was calculated with the 2-ΔΔCt method.

### Dual-luciferase reporter gene assay

Two DNA oligonucleotides that carry the complementary mature miRNA gene sequence were synthesized in BGI. Oligo sequences of the four eccMIRs are listed in Supplemental Table S10. The two complementary oligos were annealed to form short dsDNA with sticky ends and were cloned into the 3’UTR of the Renilla luciferase gene in the psiCHECK2 vector (Promega). The successful insertion of the miRNA-target sequence in the psiCHECK2 plasmid was confirmed by Sanger sequencing. Then the effects of synthetic eccDNAs on the relative expression level of Renilla luciferase were detected by the Dual-luciferase Reporter Assay System (Promega) following the manufacturer’s instruction.

### Cell proliferation assays (CCK8, clone formation and EdU assays)

The phenotype assays of MGC803 cells were performed after transfected with eccDNA for 48 h. For CCK8 testing, approximately transfected 2 × 103 cells in 100 μl were incubated in quadruplicate in 96-well plates. At 0, 1, 2, 3 and 4 day, the CCK-8 reagent (Meilunbio) was added to each well and incubated at 37 °C for 2 h. Absorbance at 450 nm was recorded by the Synergy H1M Multimode microplate reader (BioTek).

For formation analysis, approximately transfected 1 × 103 cells in 3 ml were incubated in quadruplicate in 6-well plates. Cells were stained by crystal violet (Meilunbio) followed by incubating for two weeks. Image J software (version 1.8.0; National Institutes of Health) was used for calculating the cell cluster each well.

For EdU assay, approximately transfected 8 × 103 cells in 100 μl were incubated in quadruplicate in 96-well plates. Transfected cells were added with 50 mM EdU (Beyotime) and incubated for another 2 h following by incubation for 24 h at 37 °C and 5% CO_2_. Cells were then fixed with 4% paraformaldehyde, permeated with 0.5 trition and stained with Apollo Dye Solution for proliferating cells. DAPI was used for labeling the Nucleic acids for all cells. Three randomly selected fields were taken using a fluorescence microscope. The cell proliferation rate was calculated using Image j software.

### Flow cytometry-based apoptosis detection

Apoptosing cells were detected by Annexin V-FITC/PI apoptosis kit (MultiSciences). In brief, transfected cells in 500 μl 1 × Binding Buffer suspension was stained with 5 μl of Annexin V-FITC or 10 μl PI solution for 15 min. Apoptosis detection was conducted by CytoFLEX (Beckman) in quadruplicate and calculated by Flowjo software ((Ashland, OR: Becton, Dickenson and Company; 2019)).

### Transwell migration and invasion assay

For migration assay, approximately transfected 5 × 10^4^ cells in 200 μl serum-free suspension was incubated in the upper chamber, and 600ul 1640 medium containing 10% FBS was supplied in the bottom chamber. After incubating for 12–24 h, Penetrating cells were fixed with 4% paraformaldehyde and stained with crystal violet. Cells were calculated with 4 randomly selected fields per well in quadruplicate (magnification 200x). For invasion analysis, chambers were incubated with Matrigel (BioCoat) for 2 h in advance. Other steps are the same as the migration assay.

### Statistical analysis

All statistical analysis was conducted using R-4.1.2 or GraphPad Prism 9. For screening of the GCT over-represented eccDNAs from the annotated eccDNA outcomes, the significance of eccDNAs in the two groups was analyzed by R-4.1.2 and assessed using Wilcoxon’s rank-sum test *P* value. All the wet lab assays were repeated for three to four times in independent experiments. For statistical analysis of data arising from the wet lab experiments, Student’s *t* test or one-way analysis of variance (ANOVA) was used as instructed in the Graphpad Prism 9 software to analyze the significant differences between groups. *P* < 0.05 was considered statistically significant.

## Results

### Workflow of the study

**Fig. 1:**
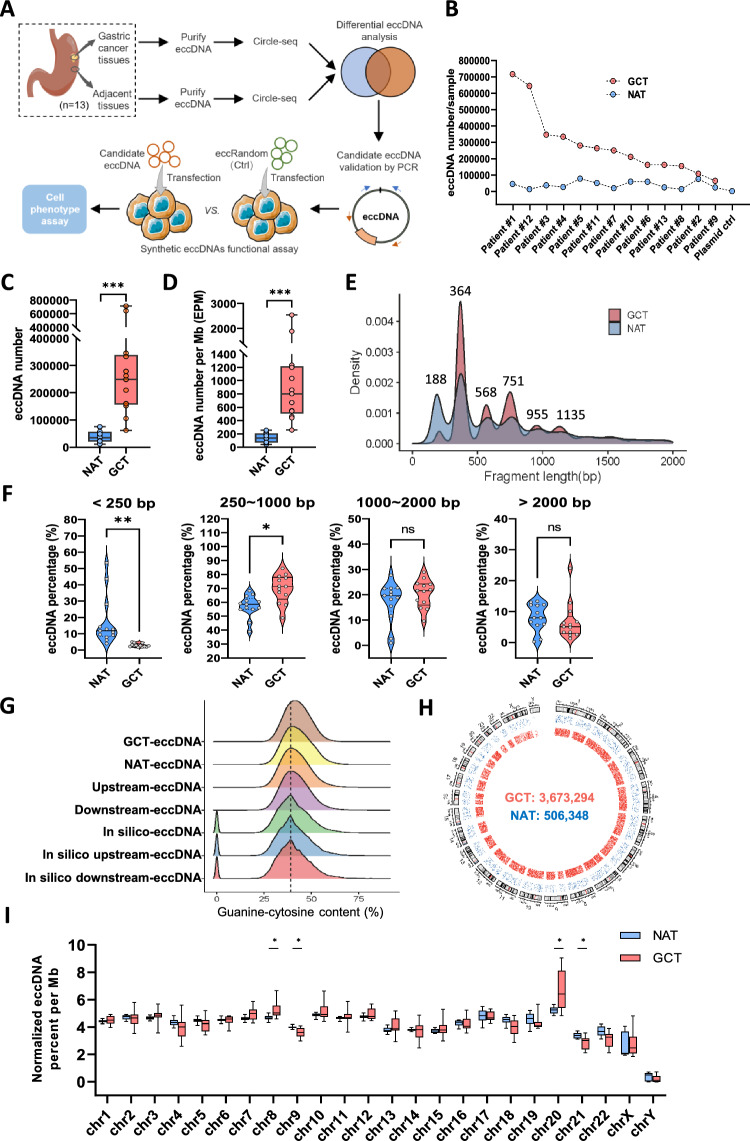
Hallmarks of eccDNA profile in gastric cancer. **A** Workflow of the study. **B** The absolute eccDNA number in the paired samples of gastric cancer patients. **C** GCT group contained significantly more eccDNAs than that of NAT group. **D** The eccDNA count per million reads mapped (EPM) of GCT group was significantly higher than that of NAT group. **E** Length distibutions of GCT- and NAT-eccDNAs. **F** Comparision of eccDNA within different length-intervals between NAT and GCT groups. **G** Guanine-cytosine content distribution of GCT, NAT, in silico eccDNAs and their upstream and downstream regions with equivalent length. **H** The genome-wide scale distributions of GCT- and NAT-eccDNAs (pool of 13 samples for each group). The red and blue dots represent the GCT derived and NAT derived eccDNAs, respectively. **I** The normalized eccDNA density (percentage of eccDNA EPM) in 24 chromosomes of GCT and NAT group

Workflow of this study is illustrated in Fig. 1A. Cancer and adjacent normal tissues were collected from thirteen GC patients. Then the eccDNAs in tissue samples were purified and the NGS reads data was deciphered utilizing Circle-seq [24, 25]. We retrieved the candidate differential eccDNAs correlated with GC by comparing the eccDNA profile of GCT with NAT. The two structural features of eccDNA, junction site and intramolecular DNA region, were confirmed by outward (blue arrows) and inward (orange arrows) PCR and Sanger sequencing. The intramolecular DNA region is defined as portion of the eccDNA sequence which does not contain the junction site sequence. The reference sequences of eccDNA candidates validated by PCR in this study are listed in Table S4 (for eccEnhancers) and Table S6 (for eccMIRs). To detect and validate the regulatory function of eccDNA carrying miRNA genes, the artificial circular DNAs were prepared by employing the Ligase-Assisted Minicircle Accumulation (LAMA) approach [21] and transfected into the host cells (MGC803 cell line in this study) for subsequent molecular function assay and cell phenotype assays, including the cell proliferation, apoptosis, invasion and migration.

### GCT contained more eccDNAs than that of NAT

In this study, we sequenced the eccDNA libraries of thirteen pairs of samples and obtained a median number of 127.2 and 152.4 million paired-end 150 reads for GCT (ranging from 107.51 to 161.23 million) and NAT (ranging from 124.23 to 165.35 million), respectively (Table S1). In total, a median number of 248,283 eccDNA loci (ranging from 62,498 to 712,918) were identified in GCT, while the number in NAT was 35,379 (ranging from 11,611 to 75,918). The eccDNA count in the paired tissues of each patient is shown in Fig. 1B. It shows there was huge variability in GCT-eccDNA counts between patients. In some patients, such as patient #1, #3 and #12, it was an order of magnitude larger, and in others, such as patient #2 and #9, it was barely more than NAT. eccDNA number in the GCT group was significantly more than those of NAT group (Fig. 1C). The normalized eccDNA count per million mapped reads (EPM) of GCT (ranging from 259 to 2537) was also significantly higher than that of NAT (ranging from 40 to 257) (Fig. 1D). The average eccDNA abundance (denoted as the average of EPM) in GCT was 7.25 times that of the NAT.

The length of around 85% of the eccDNA detected was less than 2 kb (Fig. S2) with six clear enriched peaks positioned at 188 bp, 364 bp, 568 bp, 751 bp, 955 bp and 1135 bp (Fig. 1E). These pronounced peaks showed an interval of around 180 bp, suggesting the nucleosomal origin of small eccDNAs. Importantly, the densities of GCT-eccDNAs around 364 bp, 568 bp, 751 bp, 955 bp and 1135 bp were higher than that of NAT, while the 188 bp peak area of GCT was much less than that of NAT (Fig. 1E). For detailed analysis and comparison of the eccDNA lengths between the two groups, we set 4 length-intervals: eccDNA fragments < 250 bp, 250 ~ 1000 bp, 1000 ~ 2000 bp and > 2000 bp. The results showed that “eccDNA fragments within 250 ~ 1000 bp” were more prevalent in GCT while “eccDNA fragments < 250 bp” were depleted. And there was a relatively comparable ratio of “eccDNA fragments ≥ 1000 bp” between the two groups (Fig. 1F). A median ratio of 71.4% eccDNAs in GCT was within 250 ~ 1000 bp in length while it was 58.6% in NAT group. Given the large number of eccDNAs detected in GCT samples, the higher ratio of “eccDNAs within 250 ~ 1000 bp” indicated that GCT contained much more and longer eccDNAs than that of NAT, suggesting the gastric cancer cells were prone to produce relatively longer eccDNAs which were capable of carrying longer functional genomic segments.

The 41.4% guanine–cytosine content of GCT-eccDNAs is higher than the guanine–cytosine content of the upstream (38.9%) or downstream (39.4%) flanking regions of detected eccDNA loci and the 38.8% guanine–cytosine content of the randomly generated in silico eccDNAs (Fig. 1G). In addition, we found the guanine–cytosine content of GCT-eccDNA (41.4%) was also higher than that of NAT (40.2%) (Fig. 1G), presenting a potential new property of eccDNAs in gastric cancer tissue. We then aligned either the GCT- or NAT-eccDNAs to the entire human genome reference and found they are likely to be derived from the genome randomly, although the eccDNA density of GCT (a total of 3,673,294 eccDNAsfrom the genome) was higher than that of NAT (a total of 506,348from the genome) (Fig. 1H) (Paired *t* test, *p* value < 0.001). We calculated the frequency of eccDNA generation from 24 chromosomes (percentage of EPM per chromosome) and observed the eccDNA-frequency variation in each chromosome of GCT sample was higher than that of NAT (Fig. 1I), especially in chr 8, 9, 20 and 21 (Multiple paired *t* test, *q* value < 0.05), reflecting the increased genomic instability of cancer cells in GC. In summary, these results revealed that the GCT produced more eccDNAs with higher guanine–cytosine content than that of NAT.

### Generation of eccDNAs in gastric cancer didn’t favor CpG islands

**Fig. 2:**
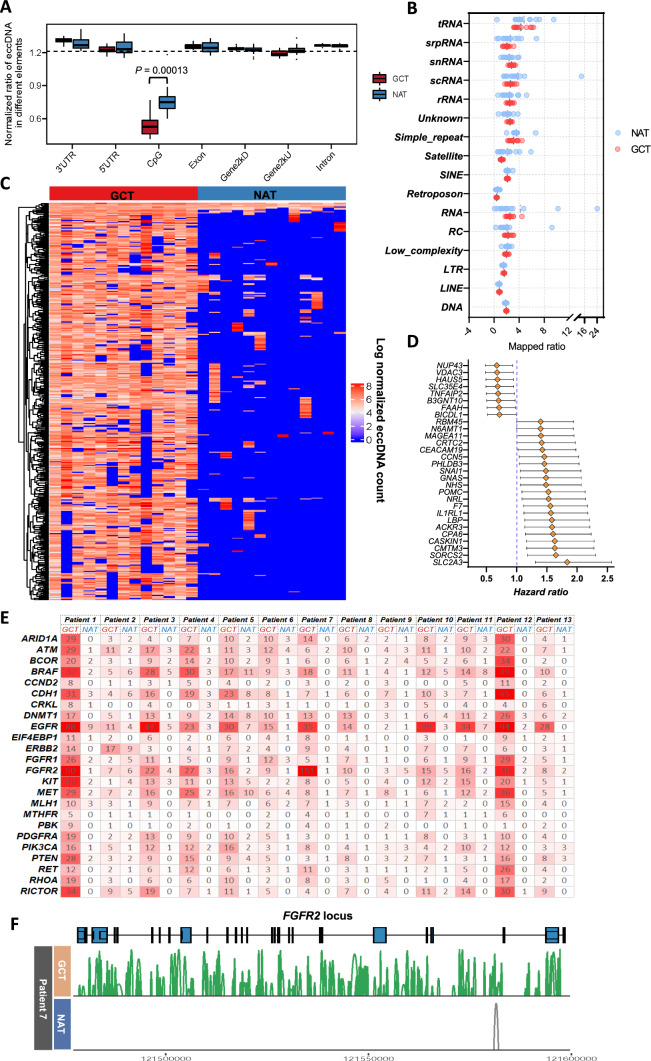
Genomic annotation of eccDNAs in GC and analysis of eccGenes. **A** Distribution of GCT- and NAT-eccDNAs in the indicated genomic regions. **B** Normalized eccDNA reads ratio mapped in specific repetitive genomic elements (median, short black line) **C** Heatmap represents the GCT differential genes with high eccDNA generation frequency compared with NAT. One row indicates one specific gene and the column indicates one sample. **D** GC prognosis-associated genes among the GCT-differential genes with high eccDNA-generation frequency. Good prognosis genes: hazard ratio < 1.0; Poor prognosis genes: hazard ratio > 1.0. **E** Copy number counts of eccDNAs derived from GC-related driver genes in the 13 individual patients. F The eccDNA browser track at the *FGFR2* gene locus in both GCT and NAT of patient #7. The connection lines represent the structure of eccDNAs detected in either GCT or NAT

We annotated all eccDNA loci with typical genomic elements and repetitive non-coding sequences. To eliminate the length bias introduced by these regions, we calculated the normalized eccDNA mapped ratio in each region with the formula: eccDNA mapped ratio = counts of eccDNA overlapping each of the genomic regions divided by the percentage of the genomic region length over the whole genome length. There was no significant difference between GCT- and NAT-eccDNAs overlapping with the six major classes of genomic elements: untranslated regions (UTR), exons, introns and 2 kb sequence upstream and downstream of gene locus (Fig. 2A). However, compared with the other six genomic elements, mapped ratio of eccDNA falling in the CpG islands was much less in both GCT and NAT (Fig. 2A). Notably, the CpG island of GCT generated significantly less eccDNA than that of NAT (*P* = 0.00013, Wilcoxon test) (Fig. 2A). Furthermore, an average of 34.77% (ranging from 32.26 to 39.14%) and 39.21% (ranging from 32.91 to 57.00%) eccDNAs contained the repetitive sequences in GCT and NAT groups, respectively, and showed no sigificant difference in these regions analyzed between the two groups (Fig. 2B). These results indicated that the CpG islands produced fewer eccDNAs than that of other gene regions in gastric cancer.

### The high abundance of eccDNAs carrying segments of GC-related driver genes may represent the DNA damage products of amplified oncogenes in tumor

The high abundance of eccDNAs derived from genic loci in somatic cells may relate to the specific physiological phenotype [24]. Thus, eccDNA harboring protein-coding gene segment (eccGene) may contribute to genome plasticity and rapid adaption. We compared the eccGene abundance (see methods) between GCT and NAT and found 412 genes were identified with a high eccDNA abundance in GCT. Despite the over representation in GCT, 3 genes were more abundant in NAT (Fig. 2C, Table S2), they were *DMRTB1*, *SORCS2* and *TST*. Among the 412 genes, 29 were related to the prognosis of gastric cancer (8 for good prognosis and 21 for bad prognosis) (Fig. 2D). We also counted the number of eccDNA derived from the GC-associated driver genes in the individual patients (Fig. 2E). The table shows that there was a high abundance of eccDNAs carrying the segments of GC-related driver genes in GCT of the individual patients. These eccDNAs may represent the DNA damage products of the relatively amplified oncogenes in tumors. Take the *FGFR2* gene in Patient 7 for example, 151 different copies of ecc*FGFR2* were detected in GCT while only 1 copy was found in NAT (Fig. 2F). These results indicated the distinct genetic difference of eccGene profiles between tumor and normal tissues.

### Over-represented eccEnhancers in GCT may contribute to development of GC

To explore the eccEnhancer profile and its possible association with GC, we annotated and compared the differential eccEnhancers between GCT and NAT. For annotation of the eccDNA harboring enhancer element in gastric cancer, we downloaded the enhancer annotation data of 6 gastric cancer-derived cell lines, including the ECC10, ECC12, MKN45, SCH, GSS and IM95m from the HACER database [26] (http://www.enhanceratlas.org/downloadv2.php), in which the enhancers were physically detected contacting with somewhere of the genomic regions by Hi-C and/or ChIA-PET. The six enhancer datasets were merged to generate one document containing 43,428 enhancers (Table S3) for annotation. These enhancers loci within the hg38 genome reference were given a series of IDs labeled from enh1 to enh43,428.

**Fig. 3:**
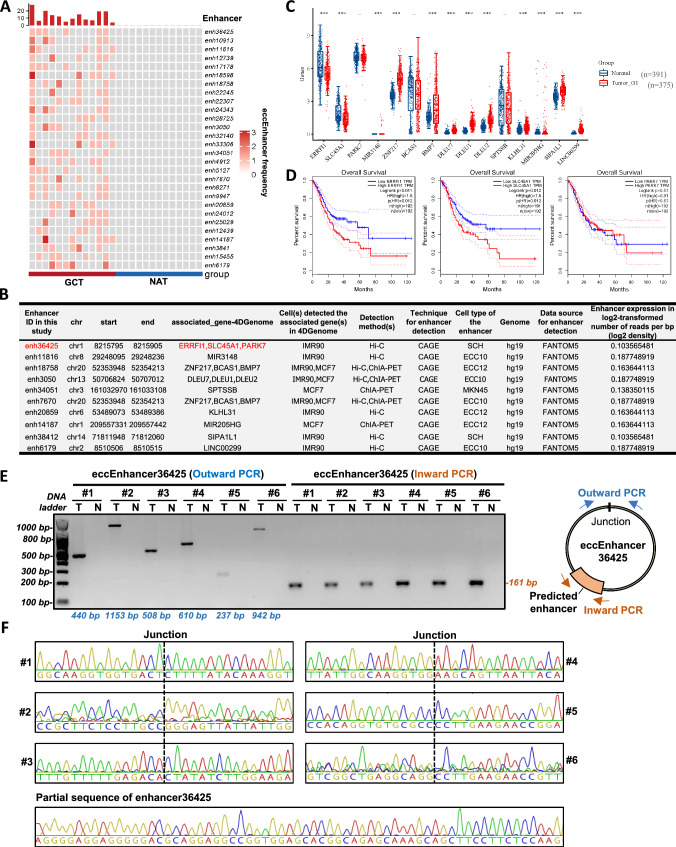
The GCT over-represented eccEnhancers may contribute to development of GC. **A** The top specific eccEnhancers found in GCT. **B** The 4Dgenome information of the top GCT over-represented enhancers in the FANTOM5 database. **C** Expression level of the enhancer-interacted target genes in the GC cohort presented in the TCGA database. **D** Overall survival analysis of the three enh36425’s target genes (*ERRFI1*, *SLC45A1* and *PARK7*) in GC cohort. **E** Outward and inward PCR of the eccEnhancer36426s found in 6 different patients. **F** Sanger sequencing results of the outward and inward-PCR products confirmed the existence of eccDNA junction sites and internal enhancer regions

In total, we identified 24,365 and 4291 eccEnhancers in GCT and NAT, respectively. The frequency of each eccEnhancer was counted in the sample of each group. Then we compared and extracted the top 28 differential eccEnhancers (Wilcoxon Rank Sum Test with *P* value < 0.05) for subsequent analysis (Fig. 3A, Table S3). We inspected the 4Dgenome information of the 28 enhancers from the FANTOM5 database and found 10 of them were associated with a set of endogenous genes (Fig. 3B), including the *ERRFI1*, *SLC45A1*, *PARK7*, *MIR3148*, *ZNF217*, *BCAS1*, *BMP7*, *DLEU7*, *DLEU1*, *DLEU2*, *SPTSSB*, *KLHL31*, *MIR205HG*, *SIPA1L1* and *LINC00299*. Interestingly, according to the data derived from The Cancer Genome Atlas program (TCGA), 12 out of the 15 enhancer-target genes (80%) were found aberrantly expressed in the gastric cancer cohort (375 tumors and 391 normal) (Fig. 3C). Among them, the target genes of enh36425, *ERRFI1* and *SLC45A1*, was negatively correlated with the prognosis of GC by overall survival (OS) rate analysis (both hazard ratio = 1.5 and *P* value = 0.012), while PARK7 was not correlated with the GC prognosis (Fig. 3D).

Furthermore, to verify the existence of the differential eccEnhancer in GCT, we performed both outward and inward PCR on the RCA products of six pairs of tissues in which the eccEnhancer36425 was found (Table S3). The size of the 6 eccEnhancer36425s ranged from 368 to 1693 bp. All the junction and internal enhancer regions of eccEnhancer36425 (labeled by #1 to #6) in GCT were validated by either outward or inward PCR (Fig. 3E). In addition, Sanger sequencing results displayed the exact sequences of these regions (Fig. 3F). Taken together, these results proved the existence of numerous small eccDNAs that carry enhancer elements in GC and suggested that they may contribute to the development of GC.

### Differential eccMIRs in GC were associated with cancer-related signaling pathways

**Fig. 4:**
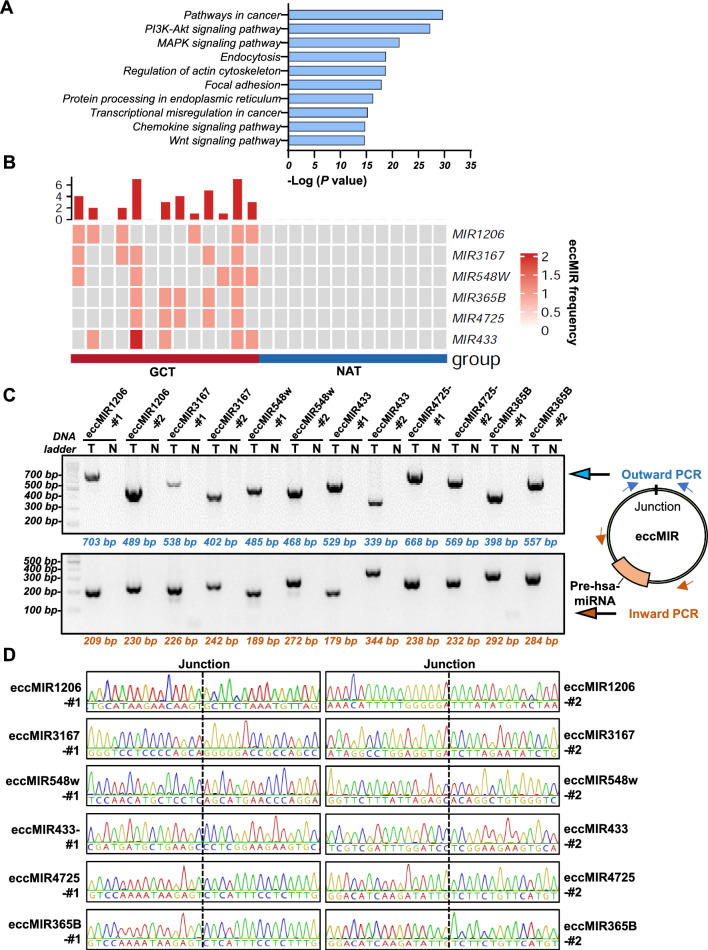
GCT over-represented eccMIRs were associated with multiple cancer-related signaling pathways. **A** KEGG analysis of the GCT over-represented eccMIRs revealed they were enriched to common cancer-associated signaling pathways, including the *PI3K-Akt*, *MAPK*, *Wnt* and so on. **B** The top six eccMIRs found in GCT. **C** Outward and inward PCR of the top 6 GCT over-represented eccMIRs (each within the two different patients labeled with #1 or #2). **D** Sanger sequencing results of the outward PCR products confirmed the existence of top 6 eccMIRs junction sites. Sanger sequencing results of inward PCR are showed in Fig. S3

Small eccDNA that carry miRNA genes (eccMIR) can produce functional miRNA molecules in host cells [21]. However, the profile of eccMIRs in cancer and their impact on cancer growth and progression were rarely studied. Based on the annotation data downloaded from miRBase, we annotated all the detected eccDNAs carrying intact hsa-pre-miRNA gene sequence (eccMIR). In total, 390 eccMIRs were identified in GCT but no eccMIR was found in the NAT. Among the 390 over-represented eccMIRs in GCT, 107 of them appeared 3–6 times in tumor samples (Table S5). To explore the potential function of these GCT over-represented eccMIRs, we performed the Kyoto Encyclopedia of Genes and Genomes (KEGG) pathway analysis using the web tool MIENTURNET (http://userver.bio.uniroma1.it/apps/mienturnet/), which allows interactive and network-based pathway analysis for a list of miRNAs [27]. Surprisingly, we found the majority of miRNAs were enriched to common cancer-associated signaling pathways, including the PI3K-Akt, MAPK, Wnt and so on (Fig. 4A). To further validate the existence of differential GCT-eccMIRs, we selected the top 6 eccMIRs (Wilcoxon Rank Sum Test with *P* value < 0.05) found in GCT (Fig. 4B) and performed PCR and Sanger Sequencing to confirm the two structural features of eccMIRs. Both outward and inward PCR verified the existence of the top 6 eccMIRs (2 eccDNAs harboring the same miRNA gene in different patients, labeled with #1 and #2, for each eccMIR validation) (Fig. 4C, Table S6) and their sequences were further confirmed by Sanger sequencing (Fig. 4D for outward PCR and Fig. S3 for inward PCR). Collectively, the above results identified the GCT over-represented eccMIRs and suggested their associations with cancer-related signaling pathways.

### GCT over-represented eccMIRs produced functional miRNA molecules in host cells

**Fig. 5:**
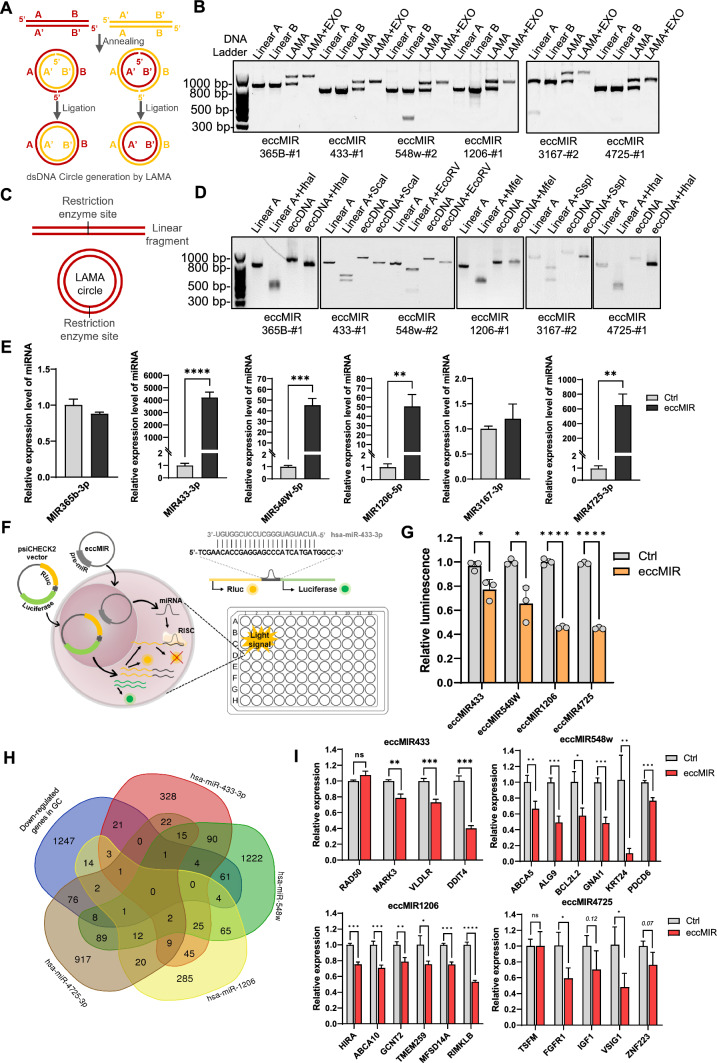
GCT over-represented eccMIRs produced functional miRNA molecules in MGC803 cells. **A** Diagram of synthetic eccDNA preparation by LAMA approach. **B** Agarose gel image of LAMA reaction products before and after exonuclease V digestion. **C** Diagram of the restriction enzyme cleave site in either linear-DNA fragment or artificial circular DNA. **D** verification of the circle structure of synthetic eccMIRs by restriction enzymes identification. **E** 4 of the top 6 eccMIRs elevated the endogenous miRNA levels in MGC803 cells. F Diagram depicting the dual-luciferase reporter gene assay of eccMIRs in MGC803 cells. the synthetic eccMIRs were co-transfected with the psiCHECK2 plasmid containing the miRNA-target sequence in 3’UTR of the Renilla luciferase gene. The eccMIR produced miRNA molecules intracellular and repressed the expression level of Renilla. Rluc: Renellia luciferase; RISC: RNA-induced silencing complex. **G** Repression of luciferase in MGC803 cells by transfection of synthetic eccMIRs. **H** The Venn plot of overlapped genes of miR433-3p, miR548w, miR1206, miR4725-3p targets with the down-regulated genes in GC. **I** The expression level of endogenous miRNA-target genes were repressed after transfection of synthetic eccMIRs in MGC803 cells. Presented by Mean and S.E. of three experiments. *Indicated *P* < 0.05, **P < 0.01, ***P < 0.001 and ****P < 0.0001

To further investigate the molecular functions of eccMIR in cells, we retrieved the top 6 eccMIRs appeared in GCT (Table S5) and synthesized each one of them detected in the samples (Table S7). The size of these eccMIRs ranged from 758 to 1194 bp. Ligase-Assisted Minicircle Accumulation (LAMA) approach was implemented to construct the target eccMIRs (Fig. 5A) and we confirmed the successful construction of artificial circular DNAs by exonuclease V digestion (Fig. 5B) and restriction enzymes identification (Fig. 5C, D). These synthetic eccMIRs were transfected into MGC803 cells and the relative expression level of either miRNA-3p or -5p produced from the corresponding eccMIR was measured by qPCR. The results showed 4 out of the 6 eccMIRs produced a high level of miRNAs in MGC803 cells (Fig. 5E).

To determine whether the eccMIRs-derived miRNAs were functional, we employed the dual-luciferase reporter gene assay system for confirmation. Dual-Luciferase reporter plasmid (psiCHECK2) containing the target sequence complementary to the miRNA in 3’UTR of Renilla was co-transfected with the synthetic eccMIR in MGC803 cells and the signals were measured 48 h after transfection (Fig. 5F). The results indicated all four eccMIRs repressed the corresponding reporter carrying miR-target UTR sequence significantly, in which the suppression efficiency ranged from 26 to 56% (Fig. 5G).

The above results suggested the GCT over-represented eccMIRs may contribute to the down-regulation of the Differentially Expressed Genes (DEGs) in GC. Therefore, we downloaded the gene list of down-regulated DEGs in GC from TCGA and obtained the predicted target-gene sets of each miRNA from miRDB (https://mirdb.org/) [28]. Gene Venn analysis by calculating the intersections of the five gene sets (down-regulated gene set in GC compared with the predicted target-gene sets of miR-433, miR-548w, miR-1206 and miR-4725) shows they share numerous genes (Fig. 5H). Then four to six of the GC-DEGs overlapped miR-target genes were selected for subsequent qPCR assay after eccMIRs transfection. The results show artificial eccMIRs down-regulated the majority of their target genes: eccMIR433 repressed hsa-miR-433 targets by up to 60%; eccMIR548w repressed hsa-miR-548w targets by up to 80%; eccMIR1206 repressed hsa-miR-1206 targets by up to 50%; eccMIR4725 repressed hsa-miR-4725 downstream genes by up to 45% (Fig. 5I). In summary, these results demonstrated GCT over-represented eccMIRs were able to produce functional miRNAs in the host cells and may contribute to the down-regulation of GC-DEGs.

### Artificial GCT over-represented eccMIRs benefited cell growth and facilitated aggressive features of host cells

Previous [21] and this study demonstrated that eccDNA carrying miRNA gene can produce functional miRNA molecules. However, their impact on cancer growth and progression is unknown. In this study, we transfected each of the four artificial eccMIRs into MGC803 cells and assessed the alterations of host cell properties by examing the cell proliferation, apoptosis, invasion, migration, DNA synthesis and cell clonogenic ability. As these eccMIRs can present simultaneously in a single patient (Fig. 4B) and they may work together to facilitate GC progression, we also tested the impact of the eccMIRs mixture on the cell phenotypes.

**Fig. 6:**
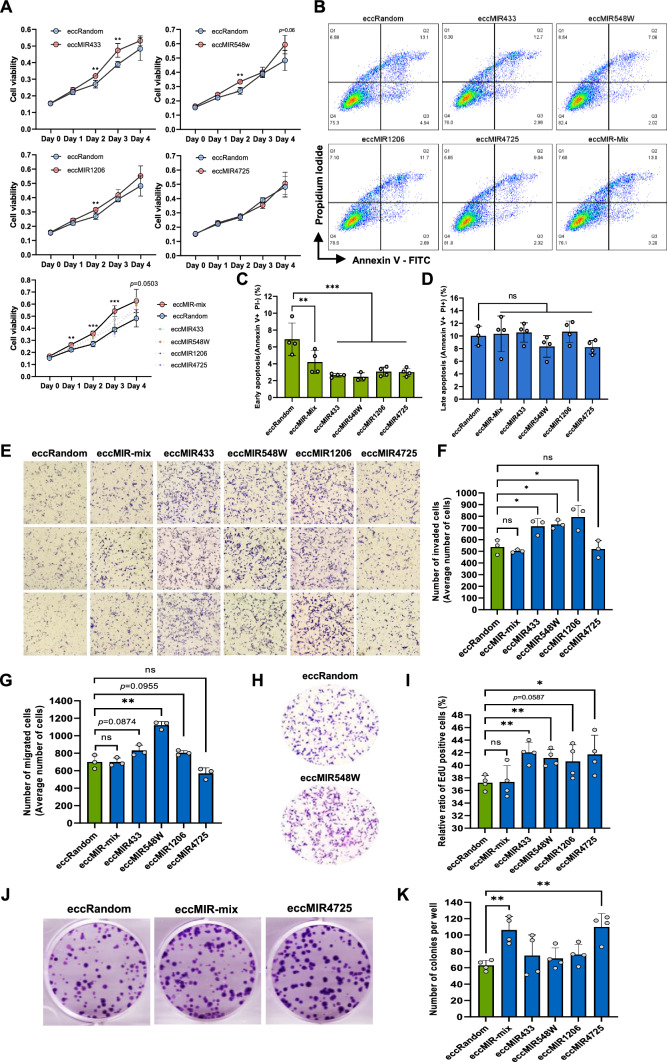
Synthetic GCT over-represented eccMIRs benefited cell growth and facilitated aggressive features of host cells. **A** Cell viability assay of MGC803 cells after sole eccMIR or eccMIR-mix transfection. **B–D** Flow cytometry-based apoptosis detection of MGC803 cells after sole eccMIR or eccMIR-mix transfection. **B** Dot plots of eccMIRs transfection groups. **C** Cells ratio of early-phase-apoptosis in each group. **D** Cells ratio of late-phase-apoptosis in each group. **E–H** Transwell migration and matrigel invasion assays of MGC803 cells after eccMIRs transfection. **E** Images represent the cells that invaded into the membrane in each group. **F** Average number of invaded cells in each group. **G** Average number of migrated cells in each group. **H** Images displayed the migrated cells in either synthetic eccRandom or eccMIR548w transfected group.** I** Relative ratio of EdU positive cells in each group after synthetic eccMIR transfection. **J** Images presented the clonogenic abilities of MGC803 cells 7 days after synthetic eccRandom, eccMIR-mix and eccMIR4725 transfection. **K** Colonies formation number in each group after synthetic eccMIRs transfection. Mean and S.E. of three or four experiments. ns indicated no significant differences; *P < 0.05, **P < 0.01 and ***P < 0.001 in a Student’s *t* test

The artificial eccMIR433, eccMIR548w and eccMIR1206 increased MGC803 cell proliferation significantly 3 days (Day 2) after transient transfection, while eccMIR4725 had no influence on cell proliferation compared with the eccRandom group (Fig. 6A). Effect of the eccMIRs on cell viability attenuated at Day 3 or Day 4, which was possibly due to the dilution or degradation of artificial eccMIRs as the cell divided. The degradation rate of eccDNA in dividing cells was similar to that of a plasmid. As shown in Fig. S4, the proportion of cells containing eccMIRs or GFP plasmid declined to ~ 40% on day 3 and decreased rapidly to ~ 10% at day 4 and 5. On the other hand, the mixture of the four artificial eccMIRs increased the cell viability significantly on day 1, 2 and 3 (Fig. 6A, eccMIR-mix), which implied the cooperative effects of multiple eccDNAs on cell growth.

The early-phase apoptosis of MGC803 cells was inhibited 2 days after artificial eccMIRs transfection (Fig. 6B, C), while the proportion of late-apoptotic cells was unaffected (Fig. 6D). Moreover, 3 of the 4 synthetic eccMIRs accelerated the invasion ability of host cells (Fig. 6E and F) while only eccMIR548w facilitated the migration rate (Fig. 6G, H). The DNA synthesis rate of host cells was also elevated significantly by eccMIR433, eccMIR548w and eccMIR4725 (Fig. 6I, Fig. S5). Finally, we observed eccMIR4725 was able to enhance the clonogenic ability of host cells and this consequence was also observed in the eccMIR-mix group, which could be imputed to the effect of eccMIR4725 but not the other three eccMIRs (Fig. S6, Fig. 6J, K). The results above demonstrated the inheritance of GCT over-represented eccMIRs benefited host cells’ growth, survival and progression.

## Discussion

Focal amplification of intact oncogene on extrachromosomal DNA has been reported to be closely related to cancer progression [2, 3, 5, 7]. Compared with the mega-base-pairs ecDNA carrying full-length gene, the role of thousands of relatively small-size eccDNAs which carry random genomic segments in cancer has not been studied as extensively. Here, we found the GCT contained significantly more eccDNAs than that of the NAT. Comprehensive analysis of the GCT over-represented eccDNAs carrying gene segments, enhancer elements and miRNA genes suggested they may contribute to tumorigenesis and development of GC. Further experimental assays demonstrated the GCT over-represented eccDNA that carry miRNA gene could produce functional miRNA molecules in host cells and promoted cell growth, survival and progression.

The large-size ecDNA could be found in almost half of the human cancers, while it is rarely seen in normal cells [3]. Similarly, it is not surprising to find the GCT contained more eccDNAs than that of the matched NAT in this study. Although the precise mechanism for eccDNA formation is enigmatic, it has been widely accepted it is associated with genomic instability related to breakage-fusion-bridge (BFB), chromothripsis, DNA damage or translocation [1, 20, 29]. Moreover, we have previously proved the two double-stranded breaks (DSBs) generated by CRISPR/Cas9 in the same chromosome can lead to the formation of eccDNAs [30, 31]. Thus, the existence of a large number of eccDNAs in GCT reflected a high degree of genomic instability and heterogeneity of cancer. These eccDNAs, usually carrying functional genomic segments, are likely to serve as a genetic reservoir for the rapid adaption and cooperative evolution of cancer.

It has been widely observed that the genome-wide eccDNAs in various samples are guanine–cytosine -rich compared with the average genomic guanine–cytosine content [18, 22, 23, 32], suggesting the generation of eccDNA from the cellular genome is not random [18]. The guanine–cytosine -rich DNA is well known as it is positively correlated with the evolutionary rates of bacteria [33] and may provide selective advantages to the earth’s biota by favoring more complex gene regulation [34]. Thus, we speculate the high guanine–cytosine compositions in cancerous eccDNAs may play a significant role during the cancer progression process. The higher guanine–cytosine content of GCT-eccDNAs compared with NAT (Fig. 1E) may also hint at this conjecture. On the other hand, the mechanism for the low CpG island compositions (Fig. 2A) of GCT-eccDNAs may be another story. Interestingly, eccDNAs derived from the normal tissues, plasma and urine were enriched in CpG islands [18, 23, 32, 35]. Nonetheless, our finding is consistent with that found in esophageal squamous cell carcinoma, where the authors reported the relatively lower CpG island origins of cancerous eccDNAs [16]. It has been reported that the CpG island loci in gastric cancer were aberrantly hypermethylated [36, 37]. Therefore, we speculated the CpG islands derived GCT-eccDNAs were prone to be methylated and had low transcription activity. These silenced eccDNAs are likely to be “useless” to cancer cells, and thus tend to be degraded and account for a low proportion within the cancerous eccDNA population. Nevertheless, more studies are needed to support this postulation.

The somatic genome derived eccDNAs have presented the potential to influence phenotypes by altering the gene copy numbers and transcribing intact or truncated genes [24]. A typical story is that the muscle-specific transcribed gene, *TTN (encodes* titin protein*),* produced a high number of eccDNAs and matched transcripts in physically active persons but not in lifelong sedentary persons [24]. These eccDNAs might be functional, or they may just represent the DNA damage products of their amplified host genes. In this study, we found a cluster of “hot-spot” genes where the eccDNAs were generated abundantly. These eccGenes possess the potential to transcribe full-length or truncated genes and work together to provide adaptive advantages to cancer cells. Meanwhile, we also found high copy numbers of eccDNAs originate from gastric cancer-related driver genes in the individual patients (Fig. 2E, F). These eccDNAs may also represent the DNA damage products of the relatively amplified oncogenes in tumors.Likewise, there was a high abundance of eccDNAs carrying intact enhancer elements in the GCT. These circular and mobile enhancers can contact with chromosomal DNA or form eccDNA-hubs and result in the activation of genome-wide transcription [4, 8]. Our analysis implied the GCT over-represented eccEnhancers may contribute to the aberrant expression of GC-DEGs and development of GC (Fig. 3B–D).

The limited size of small eccDNAs hamper them to carry the full-length protein-coding genes, but they are long enough to harbor intact miRNA gene. It has been proved that eccMIRs enable high expression of functional miRNA molecules both in vitro and in vivo [21]. In this study, signaling pathway analysis revealed these GCT over-represented eccMIRs were involved in cancer-related pathways, suggesting they play a nonnegligible role in tumorigenesis and cancer evolution. This was further confirmed by functional validation of synthetic eccMIRs (Fig. 5) and tests of their impacts on phenotypes of host cells (Fig. 6). Our study demonstrated again the molecular role of eccMIRs that they can produce functional miRNAs independent of an exogenous promoter. Moreover, we found they enhanced host cell proliferation and aggressive features. And it appeared that eccMIR transfection had only a temporary facilitation effect on host cells since only one to three cell cycles benefitted from the eccMIR transfection (Fig. 6A).

**Fig. 7:**
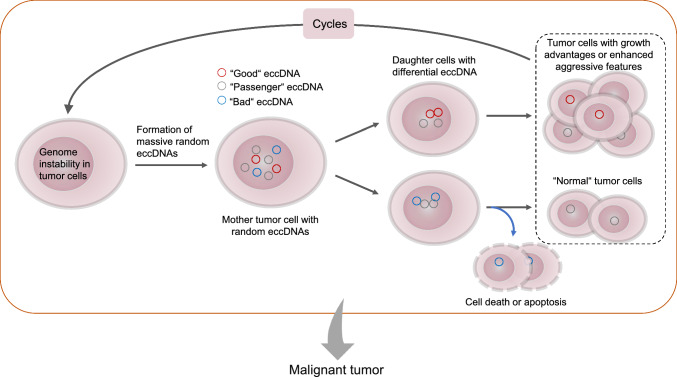
Diagram depicting the theoretical hypothesis that how the small-size eccDNAs accelerate the cancer progression. Tumor cells with instable genome can generate massive small-size eccDNAs carry random genomic segments which will be segregated randomly to daughter cells as the cell division. Host cells harboring “good” eccDNAs obtain growth advantages or increased aggressive features, whereas the inheritance of “bad” eccDNAs accelerates cell apoptosis or induces cell death. Silenced eccDNAs with no function (“passenger” eccDNA) have no effect on the host cells. This cyclic process may drive cells to evolve to malignant cells

According to the observations in this study, we propose the theoretical hypothesis of how the eccDNAs evolve cooperatively with cancer cells and facilitate cancer progression in the context of genomic instability (Fig. 7). The cancer cells produce massive eccDNAs carrying random genomic segments. Among them, there are “good” eccDNAs which can provide adaptive advantages to host cells and “bad” eccDNAs which will lead to apoptosis or cell death. The silenced or nonfunctional eccDNAs are entitled to “passenger” eccDNAs. These eccDNAs will be segregated randomly to daughter cells as cell division. The host cells which have “good” eccDNAs may grow faster or develop tolerance to environmental stress, while the cells harboring “bad” eccDNAs enter into apoptosis or cell death process. Cells containing “passenger” eccDNA undergo “normal” growth. This cyclic process helps cancer cells to overcome environmental impacts such as target treatment or immune attack, and eventually evolve to malignant tumors. Nevertheless, as few studies reported the function of small-size eccDNAs in cancers and due to the limitation of this work, further experimental and computational studies are required to substantiate this hypothesis.

In conclusion, our study revealed that gastric cancer tissues contained a large number of eccDNAs with distinct features. Comprehensive analysis of the GCT over-represented eccDNA carrying functional genomic segments suggested they were related to tumorigenesis and prognosis of GC. Moreover, the GCT over-represented eccMIRs were able to produce functional miRNA molecules and facilitate cancer progression by promoting cell proliferation and aggressive features. Overall, our study suggested that the cancerous small eccDNAs may serve as a genetic reservoir for genome plasticity and the rapid adaptive evolution of cancer. Therefore, blocking the pathways for eccDNA generation may provide a potential and novel strategy for the treatment of GC with an aberrant high level of eccDNA production.

## Supplementary Information

Below is the link to the electronic supplementary material.

**Fig. S1 **Quality control of the purified eccDNAs. **A** Standard PCR was performed on the Cox5b gene (internal linear control) which was absent from the eccDNA and a 7 kb linear-DNA fragment (external control) amplified from a plasmid, to evaluate the removal of linear DNA. + indicated the positive control (Genomic DNA as PCR template); - indicated the negative control (ddH_2_O as the PCR template). **B** Standard PCR was performed on the two spike-in plasmids to confirm the retention of circular DNAs in purified eccDNAs. + indicated the positive control in which the plasmids as the PCR template; - indicated the negative control (genomic DNA without spike-in plasmids); NTC: non template control (PPTX 513 KB)

**Fig. S2 **Percentage of eccDNAs with different size range in each sample (PPTX 160 KB)

**Fig. S3 **Sanger sequencing results of the eccMIRs’ inward-PCR products (PPTX 682 KB)

**Fig. S4 **Persist time curve of synthetic eccMIRs and GFP-plasmid in MGC803 cells. The copy number of each circular DNA was detected by qPCR on the junction site of eccMIR or the internal region of GFP-plasmid (PPTX 174 KB)

**Fig. S5 **EdU assay of MGC803 cells after synthetic eccMIRs transfection (PPTX 737 KB)

**Fig. S6 **Images of clone formation assay of MGC803 cells after synthetic eccMIRs transfection (PPTX 808 KB)

**Table S1 **Information of eccDNA NGS raw_data (XLSX 16 KB)

**Table S2 **Genes with high frequency of eccDNA generation (XLSX 47 KB)

**Table S3** Information of candidate eccEnhancers (XLSX 5226 KB)

**Table S4** Primers for eccEnhancer36425 PCR validation (XLSX 12 KB)

**Table S5** Information of GCT over-represented eccMIRs (XLSX 43 KB)

**Table S6** Primers for PCR validation of GCT over-represented eccMIRs (XLSX 17 KB)

**Table S7** Selected eccMIRs and the linear-DNA fragments for eccMIRs synthesis (LAMA) (XLSX 16 KB)

**Table S8** Primers for miRNA RT-PCR and qPCR (XLSX 11 KB)

**Table S9** Primers for qPCR assay of eccMIRs target genes (XLSX 12 KB)

**Table S10** DNA oligos for construction of psiCHECK2-UTR vector (XLSX 13 KB)

## Data Availability

The eccDNA sequencing data have been deposited in the genome sequence archive of the Beijing Institute of Genomics, National Center for Bioinformation, Chinese Academy of Science. The accession numbers for the eccDNA sequencing data in this study is HRA003499.

## Abbreviations

CPM: EccGene counts per million mapped reads
DEG: Differentially Expressed Gene
EccDNA: Extrachromosomal circular DNA
EcDNA: Large size extrachromosomal DNA carrying intact oncogene
EccGene: EccDNA carrying gene segment
EccEnhancer: EccDNA carrying enhancer element
EccMIR: EccDNA carrying miRNA gene
EPM: EccDNA count per million mapped reads
GC: Gastric cancer
GCT: Gastric cancer tissue
HMW: High-molecular-weight
LAMA: Ligase-Assisted Minicircle Accumulation
NAT: Normal adjacent tissue
NGS: Next-Generation Sequencing
RCA: Rolling Circle Amplification
TCGA: The Cancer Genome Atlas program
UTR: Untranslated regions
