# Supplementary material for: Genome-wide characterization of extrachromosomal circular DNA in gastric cancer and its potential role in carcinogenesis and cancer progression
Source: Cell Mol Life Sci. 2023 Jun 27;80(7):191. doi: 10.1007/s00018-023-04838-0 (PMC10300174; doi:10.1007/s00018-023-04838-0)
Supplement: Supplementary file 1 — Fig. S1 Quality control of the purified eccDNAs. A Standard PCR was performed on the Cox5b gene (internal linear control) which was absent from the eccDNA and a 7 kb linear-DNA fragment (external control) amplified from a plasmid, to evaluate the removal of linear DNA. + indicated the positive control (Genomic DNA as PCR template); - indicated the negative control (ddH2O as the PCR template). B Standard PCR was performed on the two spike-in plasmids to confirm the retention of circular DNAs in purified eccDNAs. + indicated the positive control in which the plasmids as the PCR template; - indicated the negative control (genomic DNA without spike-in plasmids); NTC: non template control (PPTX 513 KB) [file 18_2023_4838_MOESM1_ESM.pptx]

## Slide 1
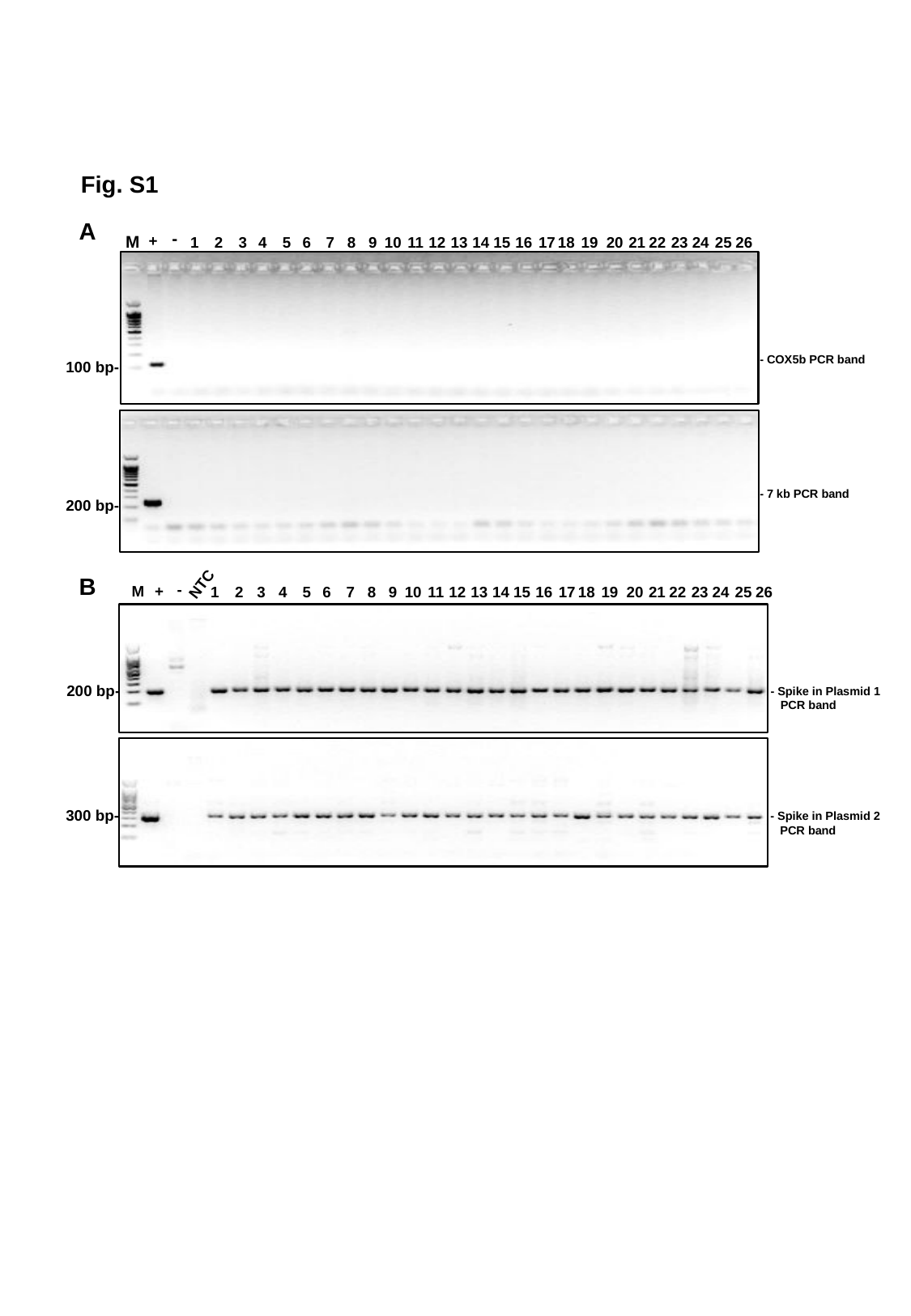

Fig. S1
A
-
M
+
1
2
3
4
5
6
7
8
9
10
11
12
13
14
15
16
17
18
19
20
21
22
23
24
25
26
- COX5b PCR band
100 bp-
- 7 kb PCR band
200 bp-
NTC
B
-
M
+
1
2
3
4
5
6
7
8
9
10
11
12
13
14
15
16
17
18
19
20
21
22
23
24
25
26
200 bp-
300 bp-
- Spike in Plasmid 1
 PCR band
- Spike in Plasmid 2
 PCR band
