# Supplementary material for: Genome-wide characterization of extrachromosomal circular DNA in gastric cancer and its potential role in carcinogenesis and cancer progression
Source: Cell Mol Life Sci. 2023 Jun 27;80(7):191. doi: 10.1007/s00018-023-04838-0 (PMC10300174; doi:10.1007/s00018-023-04838-0)
Supplement: Supplementary file 2 — Fig. S2 Percentage of eccDNAs with different size range in each sample (PPTX 160 KB) [file 18_2023_4838_MOESM2_ESM.pptx]

## Slide 1
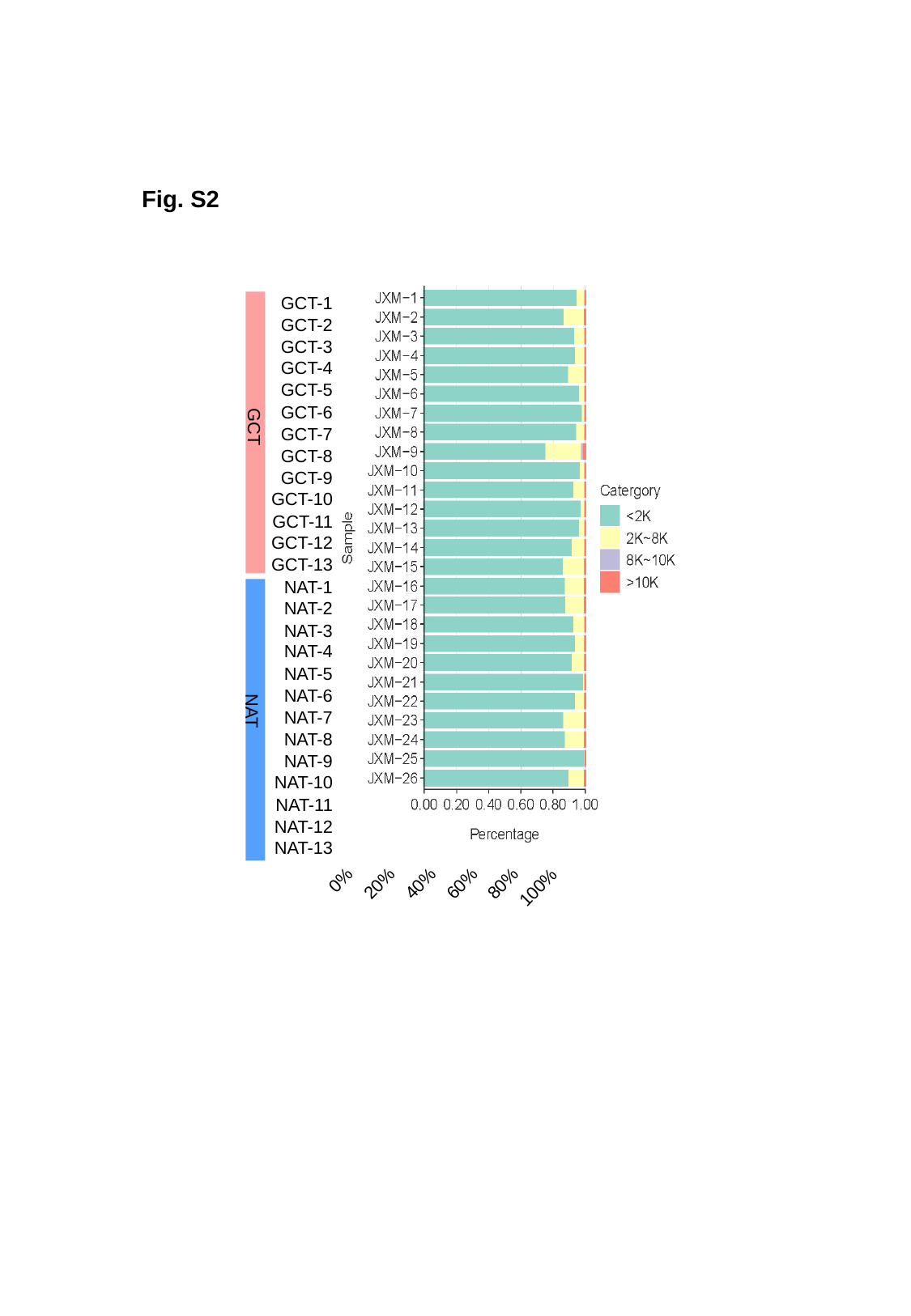

Fig. S2
GCT-1
GCT-2
GCT-3
GCT-4
GCT-5
GCT-6
GCT-7
GCT-8
GCT-9
GCT-10
GCT-11
GCT-12
GCT-13
GCT
NAT-1
NAT-2
NAT-3
NAT-4
NAT-5
NAT-6
NAT-7
NAT-8
NAT-9
NAT-10
NAT-11
NAT-12
NAT-13
NAT
0%
20%
40%
60%
80%
100%
