# Supplementary material for: Genome-wide characterization of extrachromosomal circular DNA in gastric cancer and its potential role in carcinogenesis and cancer progression
Source: Cell Mol Life Sci. 2023 Jun 27;80(7):191. doi: 10.1007/s00018-023-04838-0 (PMC10300174; doi:10.1007/s00018-023-04838-0)
Supplement: Supplementary file 3 — Fig. S3 Sanger sequencing results of the eccMIRs’ inward-PCR products (PPTX 682 KB) [file 18_2023_4838_MOESM3_ESM.pptx]

## Slide 1
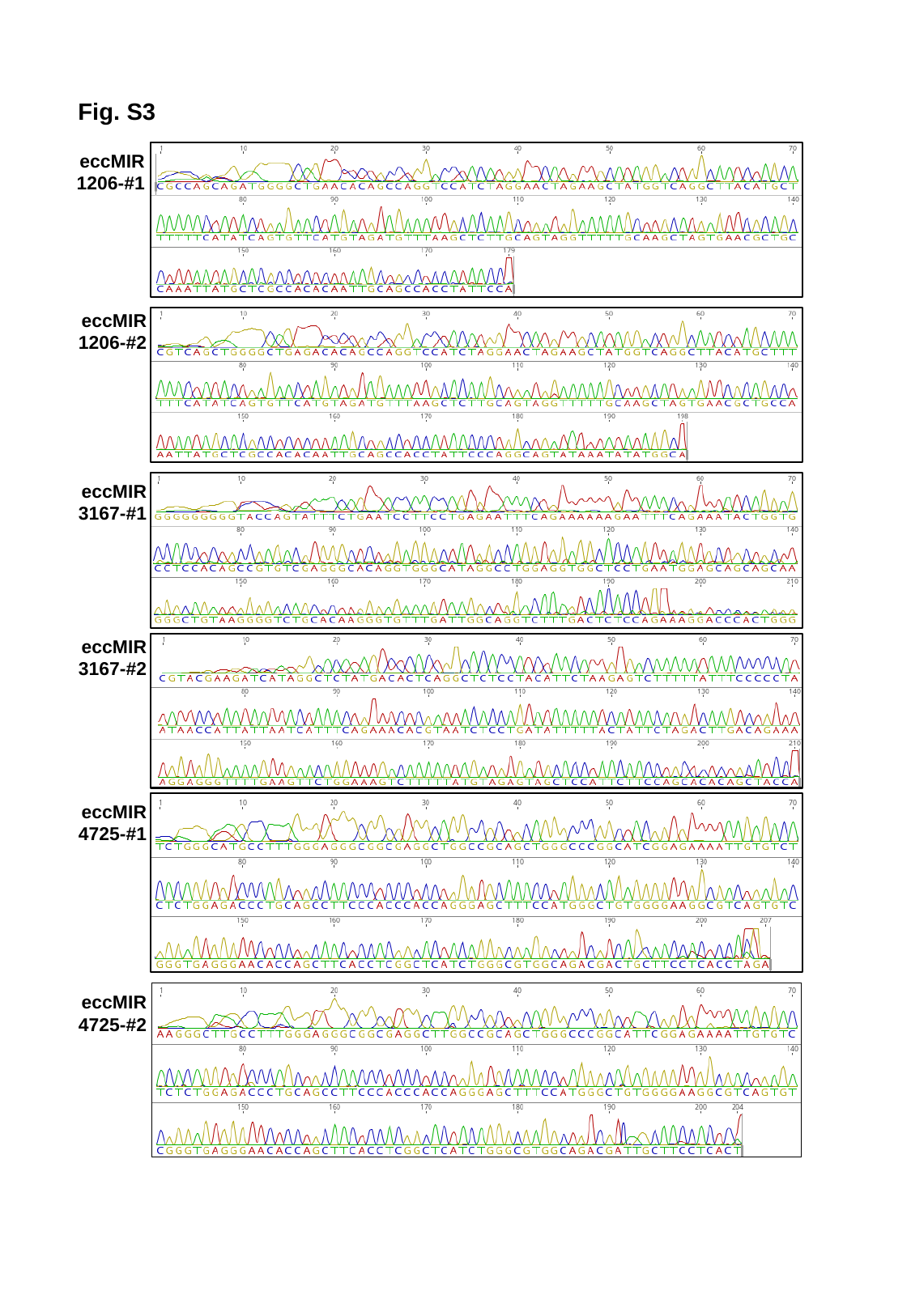

Fig. S3
eccMIR1206-#1
eccMIR1206-#2
eccMIR3167-#1
eccMIR3167-#2
eccMIR4725-#1
eccMIR4725-#2

## Slide 2
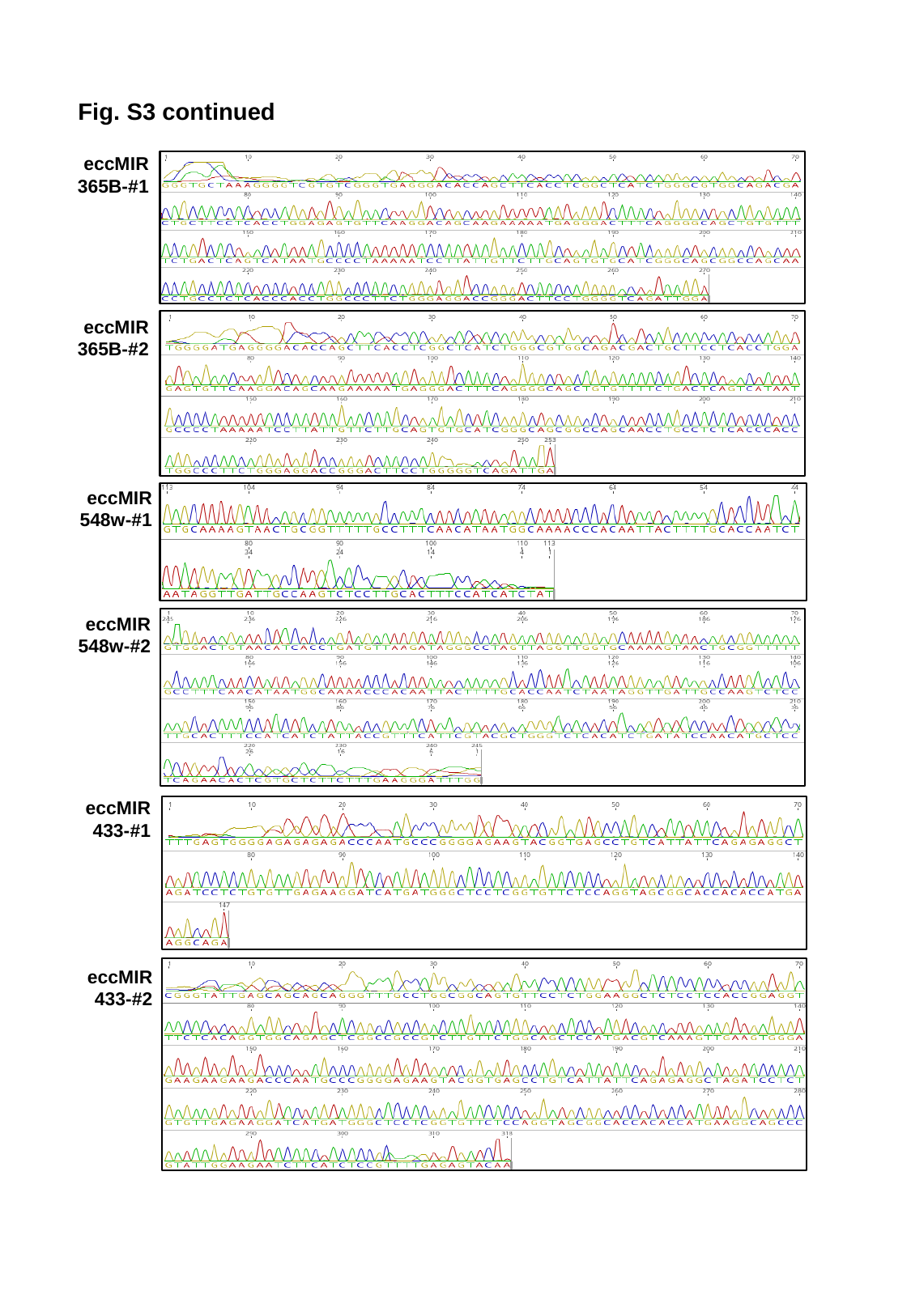

Fig. S3 continued
eccMIR
365B-#1
eccMIR
365B-#2
eccMIR
548w-#1
eccMIR
548w-#2
eccMIR
433-#1
eccMIR
433-#2
