# Supplementary figures and images for: Genome-wide characterization of extrachromosomal circular DNA in gastric cancer and its potential role in carcinogenesis and cancer progression
Source: Cell Mol Life Sci. 2023 Jun 27;80(7):191. doi: 10.1007/s00018-023-04838-0 (PMC10300174; doi:10.1007/s00018-023-04838-0)

## Slide 1
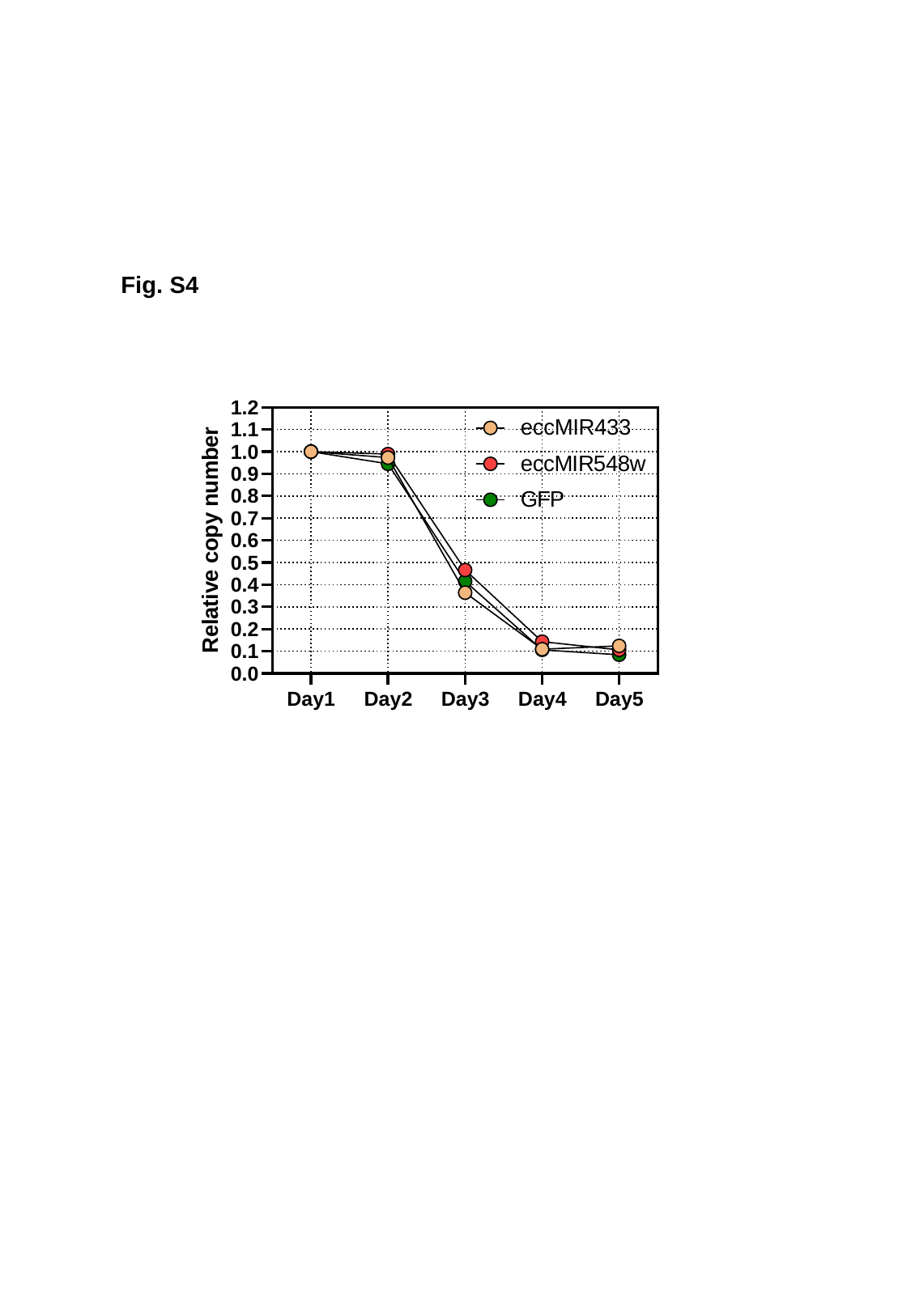

Fig. S4

Supplement: Supplementary file 4 — Fig. S4 Persist time curve of synthetic eccMIRs and GFP-plasmid in MGC803 cells. The copy number of each circular DNA was detected by qPCR on the junction site of eccMIR or the internal region of GFP-plasmid (PPTX 174 KB) [file 18_2023_4838_MOESM4_ESM.pptx]

## Slide 1
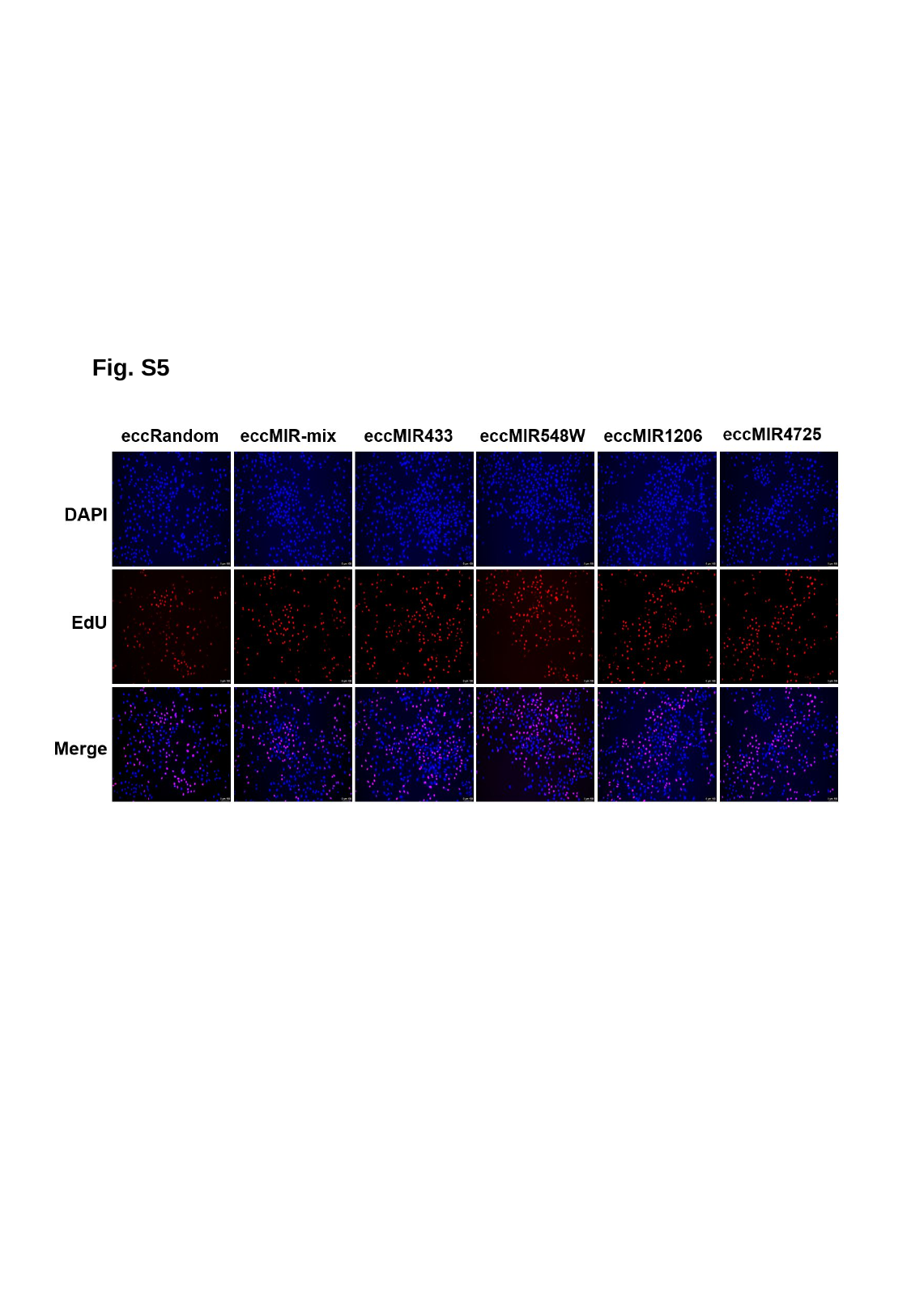

Fig. S5

Supplement: Supplementary file 5 — Fig. S5 EdU assay of MGC803 cells after synthetic eccMIRs transfection (PPTX 737 KB) [file 18_2023_4838_MOESM5_ESM.pptx]

## Slide 1
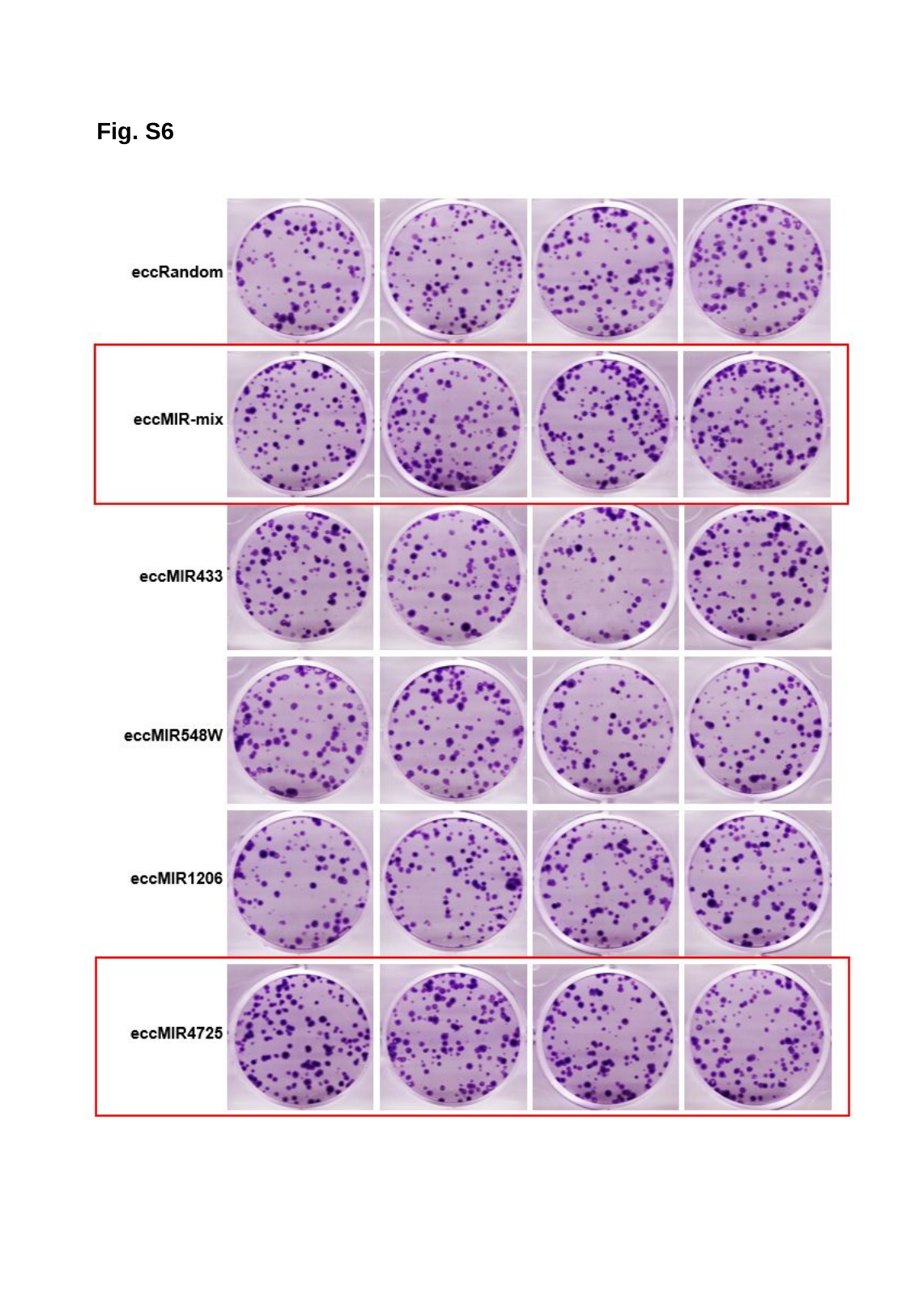

Fig. S6

Supplement: Supplementary file 6 — Fig. S6 Images of clone formation assay of MGC803 cells after synthetic eccMIRs transfection (PPTX 808 KB) [file 18_2023_4838_MOESM6_ESM.pptx]
